# Preservation of EEG spectral power features during simultaneous EEG-fMRI

**DOI:** 10.3389/fnins.2022.951321

**Published:** 2022-12-23

**Authors:** Jonathan Gallego-Rudolf, María Corsi-Cabrera, Luis Concha, Josefina Ricardo-Garcell, Erick Pasaye-Alcaraz

**Affiliations:** ^1^Unidad de Resonancia Magnética, Instituto de Neurobiología, Universidad Nacional Autónoma de México, Querétaro, Mexico; ^2^Laboratorio de Sueño, Facultad de Psicología, Universidad Nacional Autónoma de México, Mexico City, Mexico; ^3^Unidad de Neurodesarrollo, Instituto de Neurobiología, Universidad Nacional Autónoma de México, Santiago de Querétaro, Mexico; ^4^Laboratorio de Conectividad Cerebral, Instituto de Neurobiología, Universidad Nacional Autónoma de México, Querétaro, Mexico

**Keywords:** simultaneous EEG-fMRI, ballistocardiographic artifact, EEG signal preservation, EEG-informed fMRI, EEG spectral analysis

## Abstract

**Introduction:**

Electroencephalographic (EEG) data quality is severely compromised when recorded inside the magnetic resonance (MR) environment. Here we characterized the impact of the ballistocardiographic (BCG) artifact on resting-state EEG spectral properties and compared the effectiveness of seven common BCG correction methods to preserve EEG spectral features. We also assessed if these methods retained posterior alpha power reactivity to an eyes closure-opening (EC-EO) task and compared the results from EEG-informed fMRI analysis using different BCG correction approaches.

**Method:**

Electroencephalographic data from 20 healthy young adults were recorded outside the MR environment and during simultaneous fMRI acquisition. The gradient artifact was effectively removed from EEG-fMRI acquisitions using Average Artifact Subtraction (AAS). The BCG artifact was corrected with seven methods: AAS, Optimal Basis Set (OBS), Independent Component Analysis (ICA), OBS followed by ICA, AAS followed by ICA, PROJIC-AAS and PROJIC-OBS. EEG signal preservation was assessed by comparing the spectral power of traditional frequency bands from the corrected rs-EEG-fMRI data with the data recorded outside the scanner. We then assessed the preservation of posterior alpha functional reactivity by computing the ratio between the EC and EO conditions during the EC-EO task. EEG-informed fMRI analysis of the EC-EO task was performed using alpha power-derived BOLD signal predictors obtained from the EEG signals corrected with different methods.

**Results:**

The BCG artifact caused significant distortions (increased absolute power, altered relative power) across all frequency bands. Artifact residuals/signal losses were present after applying all correction methods. The EEG reactivity to the EC-EO task was better preserved with ICA-based correction approaches, particularly when using ICA feature extraction to isolate alpha power fluctuations, which allowed to accurately predict hemodynamic signal fluctuations during the EEG-informed fMRI analysis.

**Discussion:**

Current software solutions for the BCG artifact problem offer limited efficiency to preserve the EEG spectral power properties using this particular EEG setup. The state-of-the-art approaches tested here can be further refined and should be combined with hardware implementations to better preserve EEG signal properties during simultaneous EEG-fMRI. Existing and novel BCG artifact correction methods should be validated by evaluating signal preservation of both ERPs and spontaneous EEG spectral power.

## 1 Introduction

Simultaneous Electroencephalography and functional Magnetic Resonance Imaging (EEG-fMRI) records the electrophysiological and hemodynamic correlates of human brain activity non-invasively, aiming to combine the strengths of both modalities (Huster et al., 2012; Laufs, 2012; Scrivener, 2021; Ebrahimzadeh et al., 2022). EEG measures the sum of extracellular currents generated by the synchronous activity of large populations of neurons, using electrodes attached to the subject’s scalp (Schomer and da Silva, 2011), while fMRI quantifies changes in cerebral blood oxygenation, blood flow and blood volume that result from neurovascular coupling responses mediated by astrocytes, blood vessels, and neurons and therefore, represents an indirect correlate of neuronal activity (Huettel et al., 2004; Figley and Stroman, 2011). Simultaneous EEG-fMRI recording aims to better understand the complex dynamics underlying brain function by combining EEG’s temporal resolution and fMRI’s spatial resolution (Mulert and Lemieux, 2010; Scrivener, 2021; Ebrahimzadeh et al., 2022). Simultaneous EEG-fMRI also opens the possibility of directly studying interactions between electrophysiological and hemodynamic responses (Jorge et al., 2014) which cannot be achieved when signals are recorded independently (Mulert and Lemieux, 2010; Jorge et al., 2014; Ebrahimzadeh et al., 2022).

The major challenge of simultaneous EEG-fMRI recording is the presence of artifacts that compromise the data quality of both modalities (Ives et al., 1993; Mulert and Lemieux, 2010). EEG hardware may produce distortions and signal loss in MRI due to electromagnetic noise generated by the EEG amplifier (Krakow et al., 2000), B0 field inhomogeneities produced by magnetic susceptibility of EEG electrodes (Krakow et al., 2000; Mullinger et al., 2008), and B1 field attenuation produced by EEG leads (Luo and Glover, 2011; Klein et al., 2015). Image distortions can be avoided by using adequate electrode materials and placing the EEG amplifier inside a radiofrequency containment system, efficiently preserving MRI data quality at 3T (Krakow et al., 2000; Mullinger et al., 2008; Laufs, 2012). On the other hand, the magnetic resonance (MR) environment severely compromises EEG data quality (Ives et al., 1993; Lemieux et al., 1999). Two main artifacts contaminate the EEG data during simultaneous EEG-fMRI recordings: The gradient artifact (GA) and the ballistocardiographic (BCG) artifact. The GA results from induced currents over the EEG electrodes and leads that are produced by magnetic flux variations due to the gradients switching during image acquisition (Allen et al., 2000). Since the properties of the GA depend entirely on the MR sequence, these are highly stable over time and across individuals. Therefore, Average Artifact Subtraction (AAS) approaches (Allen et al., 2000) combined with hardware synchronization between EEG and fMRI equipment (Mandelkow et al., 2006) have proven to be effective in completely removing the GA.

The BCG artifact is a large-amplitude artifact that results from the induced currents caused by cardiac related movement of the EEG sensors when the subject is inside a strong magnetic field (Allen et al., 1998; Yan et al., 2010). The largest BCG artifact peak is typically observed ∼200 milliseconds after the QRS-wave recorded on the electrocardiogram (Allen et al., 1998). The artifact mainly spans cardiac harmonic frequencies ranging between 1 and 15 Hz, overlapping with the frequency of neural oscillations captured by EEG (Debener et al., 2008). Given its large variability between and within individuals, the BCG artifact represents a major challenge for EEG-fMRI. Several signal processing tools have been developed to deal with the BCG artifact and reduce its contribution from the recordings while preserving EEG signal properties (Bullock et al., 2021; Ebrahimzadeh et al., 2022). As summarized in Bullock et al. (2021) the most popular BCG correction approaches include Average Artifact Subtraction (AAS); (Allen et al., 1998), Optimal Basis Set (OBS); (Niazy et al., 2005), Independent Component Analysis (ICA); (Srivastava et al., 2005) and the combination of these: OBS-ICA (Debener et al., 2006) and AAS-ICA (Mayeli et al., 2021). Some other methods have also been recently proposed including the PROJIC-AAS and PROJIC-OBS methods (Abreu et al., 2016). Even though there have been studies comparing these methods, most of such studies have been based on assessing artifact reduction by comparing the amplitude of the artifact waveform (Mullinger et al., 2013b; Marino et al., 2018) or the reduction of its spectral components from the EEG signals before and after applying artifact correction (Abreu et al., 2016; Bullock et al., 2021). Although assessing artifact removal is important when validating BCG correction methods, the ultimate goal is to preserve the integrity of the functional properties of EEG signals. However, there are actually less studies focusing on signal preservation than artifact reduction (Marino et al., 2018; Bullock et al., 2021). Importantly, most of these studies have focused on evaluating the preservation of event related responses obtained from task paradigms (Debener et al., 2006; Assecondi et al., 2010; Vanderperren et al., 2010), where the high number of epochs used for averaging significantly increases the signal-to-noise ratio of the signals, as compared to continuous recordings (Schomer and da Silva, 2011). With a growing number of alternatives proposed to deal with the BCG artifact, there is a tremendous need to evaluate the efficiency of these methods to also preserve the spectral properties of spontaneous EEG oscillations recorded during resting-state and task paradigms, and to address the impact of BCG artifact residuals/EEG signal loss on multimodal data analysis results (Marino et al., 2019; Bullock et al., 2021). Therefore, the aim of this work was to characterize the impact of the BCG artifact on spontaneous EEG spectral power and to compare the effectiveness of the most commonly used BCG correction methods to remove the artifact while preserving underlying EEG signals. Specifically, we evaluated the spectral profile of resting-state EEG signals recorded during EEG-fMRI before and after BCG artifact correction, as compared to the spectral power of the EEG data recorded outside the scanner. We then assessed whether the functional reactivity of posterior EEG alpha power to a simple eyes closure-opening task was preserved after BCG removal and evaluated how the choice of BCG correction method affected the results from EEG-informed fMRI analysis.

## 2 Materials and methods

### 2.1 Participants

EEG and MRI data were collected from 20 healthy male individuals (mean age = 26 years; SD = 3.8 years) who were all graduate students from the Universidad Nacional Autónoma de México, campus Juriquilla (UNAM) community. Before enrolling participants into the study, the research protocol was explained to them both verbally and *via* an informed consent form. A psychologist with experience applying neuropsychological tests (JG) administered the Spanish version of the MINI International Neuropsychiatric Interview (Sheehan et al., 1997; Ferrando et al., 1998). Only cognitively healthy individuals who did not have diagnosis of any neurological or psychiatric disease or history of substance abuse were invited to participate in the study. As a last filter, participants were asked to fill in a brief checklist to corroborate the presence of counter-indications to perform the MR protocol. Individuals that fulfilled the requirements to be included in the sample and agreed to participate in the experiment signed the consent form and were recruited for the study. This research project was conducted in accordance with the principles of the Declaration of Helsinki for experiments involving human participants and was approved by the Bioethics Committee of the Instituto de Neurobiología, UNAM.

### 2.2 EEG data acquisition

Both EEG and MRI data were acquired in a single session lasting around 2.5 h. EEG data were recorded using a GES 400 MR system equipped with a 32-channel MR-compatible EEG cap (Electrical Geodesics Inc., Eugene, OR, USA). The sampling rate was 1000 Hz and Cz was used as the reference electrode. Electrode impedances were measured before starting the outside EEG recordings and all sensors were adjusted to keep impedance values below 50 k-ohms. A silk mesh was placed over the electrode cap and bandages were used to reduce electrode movement and improve EEG data quality (Ives et al., 1993; Bénar et al., 2003). Electrocardiogram (ECG) data was recorded using MR-compatible patch electrodes. The active electrode was placed over the heart (slightly to the left of the sternum bone) and the reference electrode was placed over the medial end of the left collarbone.

Electroencephalographic data were first recorded outside the MR environment, with the participant lying down in supine position, same as inside the MR-scanner. The outside EEG recording protocol consisted of 2 min of eyes-closed resting-state (Outside rs-EEG), 2 min of eyes-open EEG (not used in this study) and 2 min of an eyes closure-opening task (Outside EEG EC-EO) consisting of 20-s blocks, starting with eyes-closed. After the outside EEG recording, the participant was taken into the MR room. Once the participant was inside the scanner, EEG leads were carefully examined in search for loops and oriented in a straight line parallel to the B0 magnetic field, to reduce EEG artifacts and the risk of radiofrequency-induced heating of the sensors (Yan et al., 2009; Chowdhury et al., 2015; Assecondi et al., 2016). Sandbags and tape were used to minimize electrode leads movement and soft pads were placed between the receiving coil and the subject’s head to reduce participant’s movement (Bénar et al., 2003; Bullock et al., 2021; Ebrahimzadeh et al., 2022). The EEG amplifier was placed next to the bed of the scanner behind the 400 Gauss magnetic field iso-intensity line, in accordance with the safety guidelines provided by the vendor. Lights and ventilation systems were turned off during the entire session, to avoid further artifacts in the EEG signal (Mullinger et al., 2013a; Nierhaus et al., 2013; Rothlübbers et al., 2015). Due to our facility regulation protocols, the helium cooling pump remained active for all of the recordings.

After ensuring the participant was feeling comfortable inside the scanner, we recorded 2 min of eyes-closed EEG (Inside rs-EEG) without image acquisition. We then began the simultaneous EEG-fMRI protocol, which consisted of 10 min of eyes-closed resting-state (rs-EEG-fMRI) and 4 min of the eyes closure-opening (EEG-fMRI EC-EO) task.

### 2.3 MRI data acquisition

Brain magnetic resonance images were obtained with a Discovery MR750 3.0T scanner (General Electric, Milwaukee, WI, USA), equipped with a 32-channel array head coil. Blood-oxygen level-dependent (BOLD) contrast images were acquired for the rs-EEG-fMRI and EEG-fMRI EC-EO conditions using an echo-planar reconstruction (spatial resolution = 4 × 4 × 4 mm^3^ voxels, TR = 2000 ms, TE = 40 ms). High resolution structural sagittal T1-weighted images (spoiled gradient-recalled sequence; resolution of 1 × 1 × 1 mm^3^ voxels; TR = 8.1 ms; and TE = 3.2 ms) were collected following the simultaneous EEG-fMRI recordings, after the EEG cap was removed from the participant’s head.

### 2.4 EEG preprocessing and BCG artifact removal

The Outside rs-EEG, Inside rs-EEG, rs-EEG-fMRI, Outside EEG EC-EO and EEG-fMRI EC-EO data were preprocessed separately following the same pipeline, with the exception of the artifact removal steps that were added to correct the gradient (rs-EEG-fMRI, EEG-fMRI EC-EO) and the BCG (inside rs-EEG, rs-EEG-fMRI, EEG-fMRI EC-EO) artifacts from the data acquired inside the MR-environment.

The GA removal was the first preprocessing step for the rs-EEG-fMRI and the EEG-fMRI EC-EO datasets and was implemented directly in the Net Station software (Electrical Geodesics Inc., Eugene, OR, USA). We applied AAS by averaging the signals aligned with the event markers automatically generated by the hardware synchronization between the EEG amplifier and the MR scanner clock, using a sliding-window consisting of 5 TR volumes to generate the template. The rest of the preprocessing for all datasets was performed using customized scripts calling EEGLAB (Delorme and Makeig, 2004) and MATLAB (The MathWorks, Inc., Natick, MA, USA) functions. EEG data from all conditions (.mff files) were imported into MATLAB following the EEGLAB data structure by using the MFFmatlabIO plugin. Only data from the eighteen 10–20 system electrodes (Fp1, Fp2, F3, F4, F7, F8, Fz, C3, C4, P3, P4, Pz, T3, T4, T5, T6, O1, O2; Cz was used as reference) were considered for the analysis. Channel locations were set using the corresponding Geodesic Sensor Net template from EEGLAB. Continuous EEG data were band-pass filtered (1–50 Hz) and then segmented into 2-s epochs. EEG signals were visually inspected, and epochs containing high amplitude artifacts related to the subject’s movements or blinks were rejected. Additionally, in the case of the Inside rs-EEG, rs-EEG-fMRI and EEG-fMRI EC-EO datasets we corrected the BCG artifact using one of seven methods: (1) AAS, (2) OBS, (3) ICA, (4) OBS followed by ICA, (5) AAS followed by ICA, (6) PROJIC-AAS, and (7) PROJIC-OBS. The detection of QRS peaks and the implementation of the AAS and OBS-based correction approaches (Iannetti et al., 2005; Niazy et al., 2005) were performed using the EEGLAB FMRIB plug-in provided by the University of Oxford Centre for Functional MRI of the Brain. A constant delay of 210 milliseconds between the cardiac event markers and the main BCG peak was assumed for all AAS and OBS-based methods, which is the default value in the FMRIB plugin (Allen et al., 1998). For OBS-based corrections, the four principal components that explained most of the artifact’s waveform variance were automatically selected and regressed-out from the data. ICA was implemented using EEGLAB’s *runica* algorithm. A variable number of artifact-related independent components (ICs) were manually selected for each subject, based on criteria suggested in previous studies (Srivastava et al., 2005; Debener et al., 2006, 2008; Iannotti et al., 2014). Specifically, we removed ICs displaying all three of the following features: (1) time-series with rhythmic peaks that followed the ECG trace, (2) increased power showing multiple peaks at cardiac-related frequencies, and (3) topographical distribution of power showing either left-right or anterior-posterior polarity inversion. PROJIC-AAS and PROJIC-OBS methods were implemented using the code provided by Abreu et al. (2016). Both methods rely on applying the same functions from the FMRIB plugin, but in this case the AAS and OBS corrections are applied on the ICs timeseries before retrieving the original EEG time series by multiplying the EEG activations * mixing matrix W^–1^, in contrast to applying the correction directly on the sensor timeseries as with the regular AAS and OBS approaches. For both PROJIC approaches, we used the recommended default parameters, with the only major difference that we used the same ICs we manually selected for the ICA approach, rather than using the PROJIC-ICA automatic selection of the BCG-related components (which failed to accurately identify the BGC-related ICs for many subjects).

### 2.5 Data analysis

#### 2.5.1 Eyes-closed resting-state EEG

To evaluate the impact of the BCG artifact on EEG spectral power and to test if resting-state EEG spectral properties could be preserved after artifact correction, we compared the absolute and relative power from the corrected rs-EEG-fMRI signals vs. the Outside rs-EEG. The first available twenty-two (minimal number of clean epochs available per subject), 2-s non-overlapping clean epochs from the Outside rs-EEG and rs-EEG-fMRI conditions for each subject were selected for quantitative analysis. We computed the fast Fourier transform of the signals and then calculated the absolute and relative power across traditional EEG frequency bands (Schomer and da Silva, 2011), defined as follows: Delta = 1–3 Hz, Theta = 4–7 Hz, Alpha = 8–12 Hz, Slow beta = 13–17 Hz, Fast beta = 18–30 Hz, and Gamma = 31–50 Hz.

To obtain a qualitative measure of the BCG artifact contribution to each frequency band and visualize the variability across subjects before and after artifact correction, we calculated the percentage change in absolute power from the rs-EEG-fMRI relative to the outside rs-EEG [(rs-EEG-fMRI power/outside rs-EEG power) *100] −100, for each electrode of each subject. For the statistical analysis, we used one-way repeated measures ANOVAs to compare the log-transformed absolute power and the relative power values of the corrected rs-EEG-fMRI and the Outside rs-EEG. Each frequency band was considered independently. Bonferroni correction for multiple comparisons was applied to the *p*-values of the *post-hoc* test between the Outside rs-EEG and the seven corrected versions of the rs-EEG-fMRI data. Adjusted *p*-vales below 0.05 were considered to be significant. To discard the contribution of GA residuals and further validate our results, we repeated our analysis using the Inside rs-EEG instead of the rs-EEG-fMRI data (Supplementary material).

In addition to the absolute and relative spectral power analysis, we tested the reliability of the estimates of the individual alpha peak frequency and alpha center of gravity from the power spectrum of the resting-state signals collected during EEG-fMRI. Following the methods and using the code provided by Corcoran et al. (2018), we employed an automated approach based on applying a Savitzky–Golay filter (Klimesch et al., 1990) to calculate each individual’s alpha peak frequency and center of gravity. We set the band-pass filter from 1 to 40 Hz and looked for the alpha peak in the range between 7 and 13 Hz. We set a value of 11 for the Savitzky–Golay filter frame width and a *k* = 5 for the polynomial order. For the statistical comparison, we used one-way repeated measures ANOVAs to compare the individual alpha peak frequency and center of gravity estimates from the corrected rs-EEG-fMRI and the Outside rs-EEG (Supplementary material). We applied Bonferroni correction to account for multiple comparisons and considered adjusted *p*-vales below 0.05 to be significant. Once again, we repeated our analysis using the Inside rs-EEG instead of the rs-EEG-fMRI data (Supplementary material).

#### 2.5.2 Eyes closure-opening task EEG data

Given that the posterior alpha power reactivity to the eyes closure-opening task is one of the most prominent and consistent features observed in human EEG recordings (Berger, 1929; Barry et al., 2007; Klimesch et al., 2007; Barry and De Blasio, 2017), we selected this task to evaluate if alpha power functional reactivity was preserved in the EEG-fMRI signals corrected using different BCG removal approaches. The first available twenty, 2-s non-overlapping EEG epochs from the eyes-closed and eyes-open blocks of the Outside EEG EC-EO and the EEG-fMRI EC-EO conditions were selected for each subject and submitted to fast Fourier transform, as implemented previously. Besides the seven BCG correction methods used before, an eighth method consisted of using ICA as a feature extraction tool (IFE), aiming to isolate alpha power activity related to the task. In this case, instead of removing the ICs associated with the BCG artifact we only retained components with a time-series that showed clear alpha activity during the EC blocks, a peak around 10 Hz in its power spectrum, and a topographical distribution showing higher alpha power in posterior electrodes. To estimate a quantitative difference between the two physiological states, we calculated a ratio by using the signal from occipital O1 and O2 electrodes and dividing the alpha power of the EC over the EO condition (EC-EO alpha power ratio). We performed the statistical analysis on the EC-EO alpha power ratio values rather than the raw eyes-open and eyes-closed alpha power values given that absolute and relative alpha power is highly variable across individuals (Shaw, 2003). To assess signal preservation, a one-way repeated measures ANOVAs was performed to compare the EC-EO alpha power ratio between the Outside EEG EC-EO and the EEG-fMRI EC-EO corrected with different methods (AAS, OBS, ICA, OBS-ICA, AAS-ICA, PROJIC-AAS, PROJIC-OBS, and IFE). As before, Bonferroni-correction for multiple comparisons was applied to the *p*-values of the *post hoc* comparisons, and adjusted *p*-values > 0.05 were considered as significant.

#### 2.5.3 EEG-informed fMRI analysis

Functional magnetic resonance imaging data preprocessing and analysis was performed using the FSL software (Jenkinson et al., 2012). Preprocessing included motion correction, slice timing (interleaved acquisition) correction, brain extraction, spatial smoothing using a Gaussian kernel (full-width-half-maximum = 6 mm), high-pass temporal filtering (cutoff frequency = 0.01 Hz), and registration to each subject’s structural image followed by spatial normalization to the Montreal Neurological Institute standard space (MNI ICBM-152 template) using linear transformations with seven and twelve degrees of freedom, respectively. EEG-informed fMRI first-level analysis was performed using alpha power fluctuations to derive a BOLD signal predictor for each subject. To generate the predictors, we selected either the O1 or O2 channel timeseries (selected on an individual basis to obtain the best available predictor) and calculated the alpha band absolute power for each 2-s epoch. The values corresponding to epochs that were eliminated due to excessive movement or eye-related artifacts were replaced using a simple interpolation method (taking the average of the previous and following epoch). The resulting time series (60 timepoints) were convolved with a gamma hemodynamic response function in the GLM tool of FSL’s FEAT to generate the BOLD signal predictors. We focused on the negative contrast, as our hypothesis was that alpha power fluctuations would be negatively correlated with BOLD signal. We first conducted this analysis on the full sample (*n* = 20), though no significant associations were found between the predictors and the BOLD signal using any method, due to some individuals that did not show any associations between the signals in the first-level analysis. We therefore repeated the analysis after removing these 5 individuals, which corresponds to the data presented here.

To assess the impact of BCG artifact residuals on preserving the EEG functional reactivity for multimodal integration, the EEG-informed fMRI analysis was repeated using the alpha power predictors derived from the same EEG signals, corrected using each method. Second-level analyses were performed using permutation-based inference (Nichols and Holmes, 2002) with threshold-free cluster enhancement to account for multiple comparisons (Smith and Nichols, 2009) as implemented by FSL’s randomize function. Group-level statistical parametric maps obtained from the EEG-informed fMRI analyses were compared with conventional fMRI analysis results, performed using the task design to build the hemodynamic response predictor.

### 2.6 Data/code availability statement

All the data used in this study is available on an open data repository: “Simultaneous EEG-fMRI dataset,” Mendeley Data, V1, doi: 10.17632/crhybxpdy6.1 (Gallego-Rudolf et al., 2022). All the preprocessing and analysis steps in this study used a combination of existing documented functions from MATLAB v.18b (The MathWorks, Inc., Natick, MA, USA) and EEGLAB v.14.1.2b (Delorme and Makeig, 2004) software packages. EEG-informed fMRI was conducted using the FSL software (Jenkinson et al., 2012). Statistical analysis was performed in R Studio, using R v.4.1.1 (R Core Team, 2022) and ggplot2 (Wickham, 2016) was used to generate the plots.

## 3 Results

### 3.1 Resting-state–BCG artifact reduction and preservation of EEG spectral features

**FIGURE 1:**
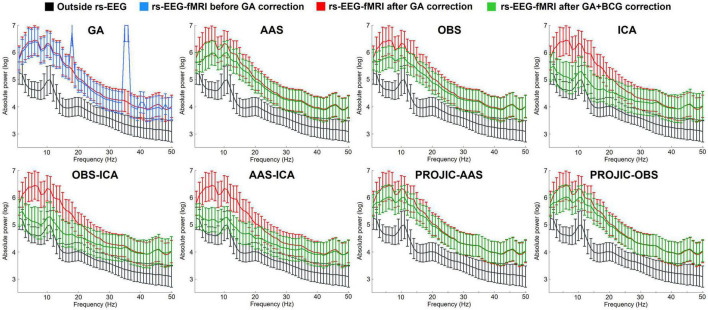
Average power spectrum (and standard deviation) computed from the resting-state eyes-closed EEG signal of all electrodes from all subjects. The first panel shows the Outside rs-EEG (black) and the rs-EEG-fMRI data before (blue) and after (red) GA correction. The rest of the panels show a comparison between the Outside rs-EEG (black line, repeated in all panels), the rs-EEG-fMRI after GA removal but before BCG artifact correction (red line, repeated in all panels) and its corrected version (green) after using one of seven BCG correction methods: Average Artifact Subtraction (AAS), Optimal Basis Set (OBS), Independent Component Analysis (ICA), OBS-ICA, AAS-ICA, PROJection onto Independent Components (PROJIC)-AAS or PROJIC-OBS. ICA-based corrections performed better in reducing the BCG artifact contribution and preserving the spectral profile of rs-EEG-fMRI signals, though power remained higher compared to the Outside rs-EEG.

Given that the GA is entirely dependent on the properties of the MR sequence, having an adequate synchronization between the EEG and MRI hardware and using the AAS approach allowed to effectively remove the GA from the signals of all participants. The first panel of Figure 1 shows the comparison between the average power spectrum across all electrodes from all subjects from the Outside rs-EEG (black), the rs-EEG-fMRI data before GA correction (blue) and the rs-EEG-fMRI data after GA and before BCG artifact correction (red). The rest of the panels from Figure 1 show the group average power spectra for the Outside rs-EEG (black, same for all panels), the rs-EEG-fMRI before BCG artifact correction (red, same for all panels) and its corrected version (green) using each BCG correction approach. The main contribution of the BCG artifact to the power spectrum can be observed as a generalized increase in spectral power, more pronounced in the theta and slow beta range (red power spectrum). In general ICA-based approaches (ICA, but specially OBS-ICA and AAS-ICA) performed better in reducing the BCG-induced absolute power increases, partially retrieving the characteristic shape of the eyes-closed rs-EEG spectrum. The number of components removed for each method (mean; SD; range) was 9.2; 1.8; 6–12 for ICA, 4.8; 1.2; 3–7 for OBS-ICA and 5.7; 1.4; 3–8 for AAS-ICA. Supplementary Figure 1 shows that similar results were obtained when calculating the power spectrum from the Inside rs-EEG, instead of the rs-EEG-fMRI data.

**FIGURE 2:**
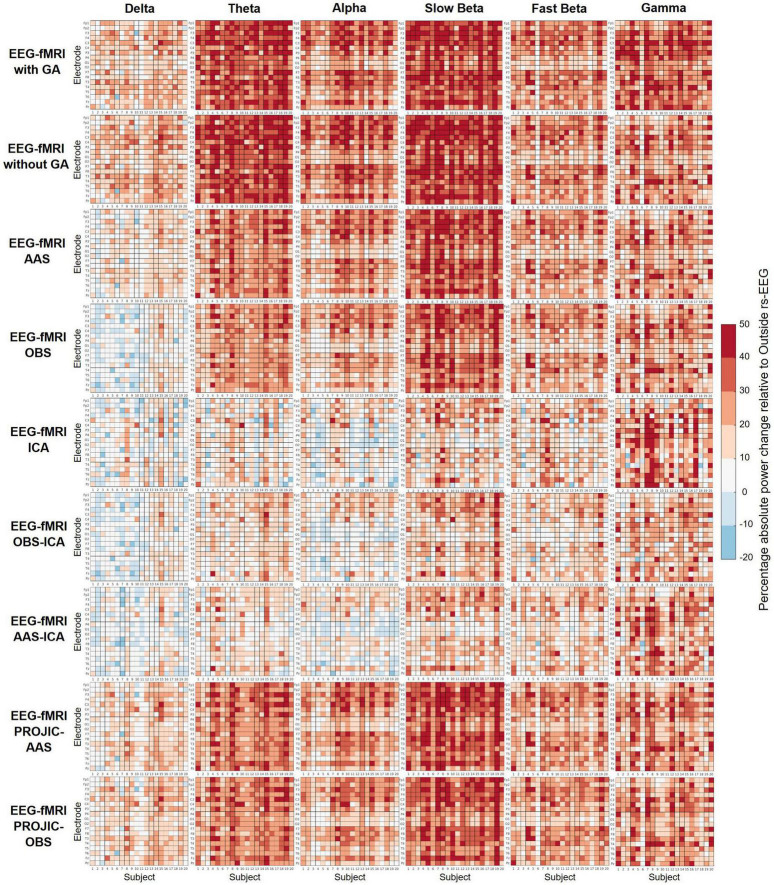
Percentage change (colorbar) in the absolute power of each frequency band of the rs-EEG-fMRI data before and after BCG artifact removal, relative to the Outside rs-EEG. Each matrix shows all electrodes (rows) for each subject (columns). A negative percentage indicates lower absolute power in the rs-EEG-fMRI compared to the outside rs-EEG. ICA-based corrections performed better in reducing the BCG artifact contribution and preserving the rs-EEG-fMRI spectral profile (especially for delta, theta, and alpha bands), though artifact residuals and/or absolute power decreases were evident for most subjects, across all frequency bands.

Figure 2 shows the percentage change in power of the rs-EEG-fMRI relative to the Outside rs-EEG, after correcting the rs-EEG-fMRI signal with different BCG removal approaches. Each matrix corresponds to a particular method and frequency band and shows all electrodes (rows) for every subject (columns). As can be observed, a considerable increase in power across all bands was present in the uncorrected rs-EEG-fMRI data, with theta and slow beta being the most affected frequency bands. Again, ICA, OBS-ICA and AAS-ICA correction methods were more efficient in suppressing the contribution of the BCG artifact, by reducing the BCG-induced power increase especially in the delta, theta, and alpha bands. However, even when using ICA–based corrections, artifact residuals remained for most subjects, particularly in the fast beta and gamma bands. Moreover, we also observed decreases in power compared to the outside rs-EEG, reflecting potential EEG signal losses produced during artifact correction.

**FIGURE 3:**
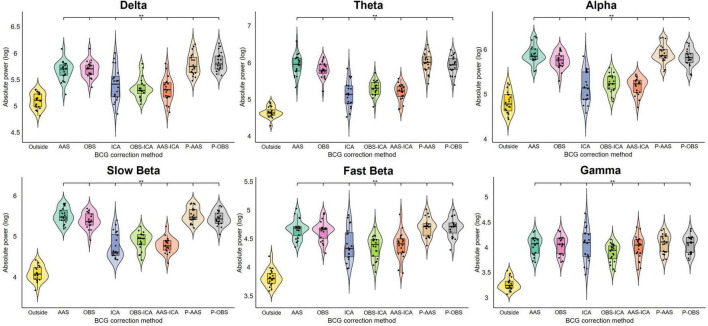
Results of the repeated measures ANOVAs comparing the average absolute power of all electrodes from all subjects between the Outside rs-EEG and the rs-EEG-fMRI data corrected using each of the seven BCG correction methods. Each frequency band was analyzed separately. The asterisks indicate significant statistical differences (*p*_adj_ < 0.05) between the corrected rs-EEG-fMRI and the Outside rs-EEG data. A generalized increase in absolute power across all frequency bands was observed for the data recorded simultaneously with fMRI, which remained significant after applying all BCG correction methods. Note that PROJIC-AAS and PROJIC-OBS were abbreviated as P-AAS and P-OBS, respectively.

The statistical analysis comparing the absolute power across the six frequency bands is shown in Figure 3. Significant statistical differences were found between the power of the Outside rs-EEG and the rs-EEG-fMRI for all frequency bands (delta *F*_3.46,65.67_ = 61.34, *p* < 0.001; theta *F*_3,56.98_ = 165.68, *p* < 0.001; alpha *F*_2.89,54.87_ = 141.33, *p* < 0.001; slow beta *F*_2.97,56.36_ = 239.32, *p* < 0.001; fast beta *F*_2.98,56.55_ = 96.57, *p* < 0.001; gamma *F*_2.58,48.94_ = 82.62, *p* < 0.001), regardless of the BCG correction method employed. Very similar results were obtained for the Inside rs-EEG data (Supplementary Figures 2, 3).

**FIGURE 4:**
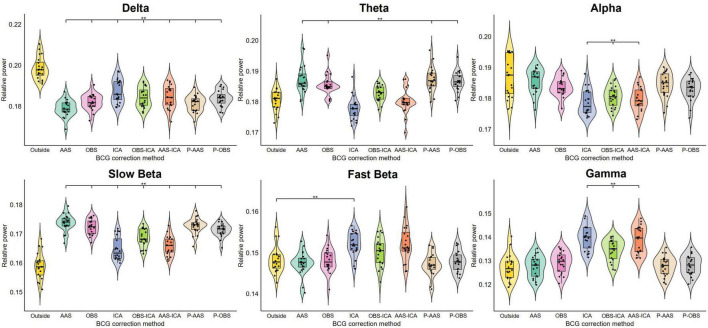
Results of the repeated measures ANOVAs comparing the average relative power of all electrodes from all subjects between the Outside rs-EEG and the rs-EEG-fMRI data corrected using each of the seven BCG correction methods. Each frequency band was analyzed separately. The asterisks indicate significant statistical differences (*p*_adj_ < 0.05) between the corrected rs-EEG-fMRI and the Outside rs-EEG data. Relative power was altered across all frequency bands for the data recorded simultaneously with fMRI. Some correction approaches rescued relative power for some frequency bands, but the overall spectral power profile remained altered across all BCG correction methods. Note that PROJIC-AAS and PROJIC-OBS were abbreviated as P-AAS and P-OBS, respectively.

Even though the BCG-induced power increase across frequency bands remained significant after artifact correction, qualitatively the rs-EEG-fMRI data showed that the individual power estimates computed after applying ICA-based correction approaches displayed a more similar distribution compared to the Outside rs-EEG values. Therefore, we also analyzed the relative power of each frequency band and compared the Outside rs-EEG vs. the corrected versions of the rs-EEG-fMRI data (Figure 4). Delta relative power was significantly decreased, while slow beta remained significantly increased across all correction methods (*F*_2.9,55.18_ = 79.33, *p* < 0.001; *F*_2.48,47.11_ = 65.84, *p* < 0.001). Theta relative power from the ICA, OBS-ICA and AAS-ICA approaches was not significantly different compared the Outside rs-EEG, which was the case for all other methods (*F*_2.79,52.93_ = 32.11, *p* < 0.001), but in contrast only these approaches showed significant differences in the alpha relative power compared to the Outside rs-EEG (*F*_1.92,36.49_ = 14.16, *p* < 0.001). The only method in which fast beta relative power was different from the Outside rs-EEG was ICA (*F*_3.03,57.56_ = 17.51, *p* < 0.001) and for gamma relative power there were significant increases observed in the ICA, OBS-ICA, and AAS-ICA approaches (*F*_2.69,51.17_ = 62.02, *p* < 0.001). Once again, we found very similar results when using the Inside rs-EEG data (Supplementary Figure 4).

The analysis of the individual alpha peak frequency and center of gravity revealed that, even with fewer electrodes with sufficient quality for the estimation of these parameters in the rs-EEG-fMRI condition (Supplementary Table 1), there were no significant differences in the estimations of the alpha peak frequency and center of gravity when comparing the corrected (and uncorrected) rs-EEG-fMRI against the Outside rs-EEG data (Supplementary Figure 5).

### 3.2 Eyes closure-opening task–preservation of EEG functional reactivity

**FIGURE 5:**
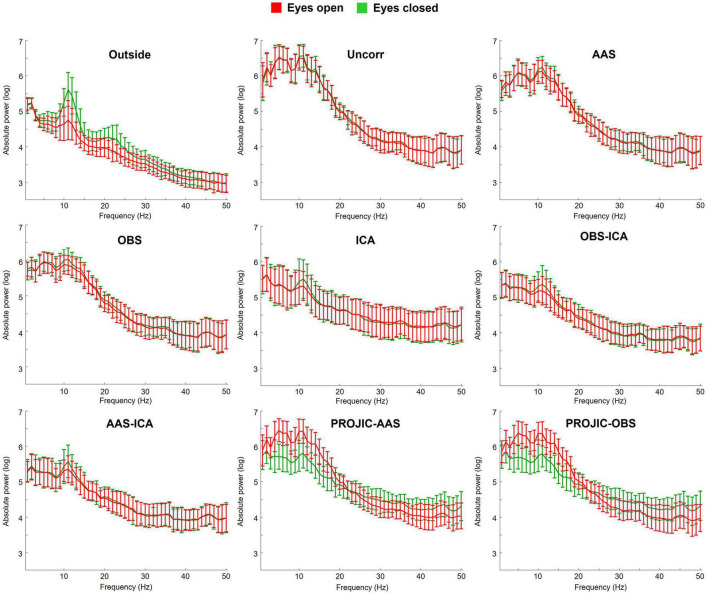
Group average power spectrum (with standard deviation) of the O1 electrode during the eyes-closed (EC; green) and eyes-open (EO; red) conditions of the EC-EO task. A comparison is shown between the spectra obtained from the Outside EEG EC-EO and the EEG-fMRI EC-EO data, before and after removing the BCG artifact with each correction method. ICA-based approaches performed better in retrieving the difference between EC and EO conditions (reflected as higher alpha power for the EC condition), though the difference was still attenuated when compared to the data acquired outside the scanner.

We then focused on evaluating if EEG functional reactivity to the EC-EO task could be preserved after BCG artifact removal. Figure 5 shows the group average power spectrum from the O1 electrode, obtained from the EEG signals collected during the eyes-closed (green) and eyes-open (red) conditions. For the Outside EEG EC-EO spectrum, a clear distinction between the two physiological states is observed as a higher-amplitude alpha power peak in the absolute power EC EEG spectrum, compared to the EO spectrum. This difference is completely masked by the BCG artifact. Although the difference between the two conditions was never as evident as for the Outside EEG EC-EO data, the use of ICA, or the combination of OBS-ICA and AAS-ICA allowed to partially retrieve the difference between EC and EO states. The number of components removed per each method (mean; SD; range) was 9.6; 1.3; 8–11 for ICA, 6.7; 1.4; 4–10 for OBS-ICA and 5.8; 1.2; 4–8 for AAS-ICA. The rest of the correction approaches failed to retrieve a clear distinction in the alpha band between the two conditions.

**FIGURE 6:**
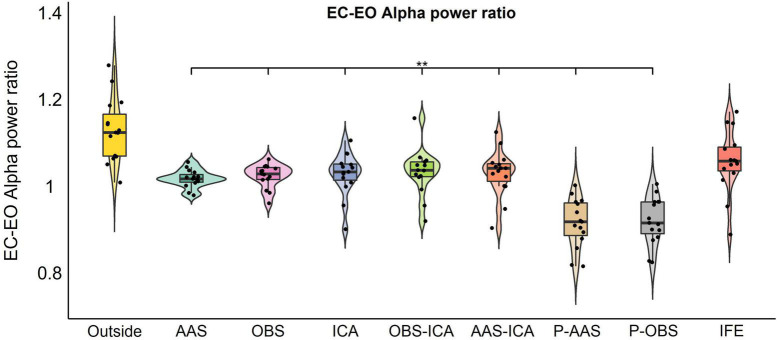
Results of the repeated measures ANOVAs comparing the eyes closure-opening (EC-EO) alpha power ratio calculated from O1 and O2 electrodes for the Outside EEG EC-EO and the EEG-fMRI EC-EO signals corrected with each BCG approach or Independent component analysis Feature Extraction (IFE). The asterisks indicate significant statistical differences (*p*_adj_ < 0.05) between the EEG-fMRI EC-EO and the Outside EEG EC-EO data. IFE was the only method that did not show significant differences in the EC-EO alpha power ratio when compared to the data recorded outside the scanner. Note that PROJIC-AAS and PROJIC-OBS were abbreviated as P-AAS and P-OBS, respectively.

This was confirmed by the statistical analysis shown in Figure 6, comparing the ratio obtained from dividing the alpha power of the EC condition by the alpha power of the EO condition. BCG artifact residuals resulted in a significant decrease in the alpha power ratio for all correction methods (*F*_2.77,38.75_ = 35.52, *p* = > 0.001). The only approach that allowed to retrieve an EC-EO power ratio that was not statistically different from the Outside EEG EC-EO data was the ICA feature extraction of the alpha power, indicating this strategy retrieved the functional reactivity of posterior alpha oscillations (Figure 6). The number of retained ICs related to alpha activity for the ICA feature extraction approach (mean; SD; range) was 2.1; 0.6; 1–3.

### 3.3 EEG-informed fMRI–impact of BCG artifact residuals on multimodal analysis

**FIGURE 7:**
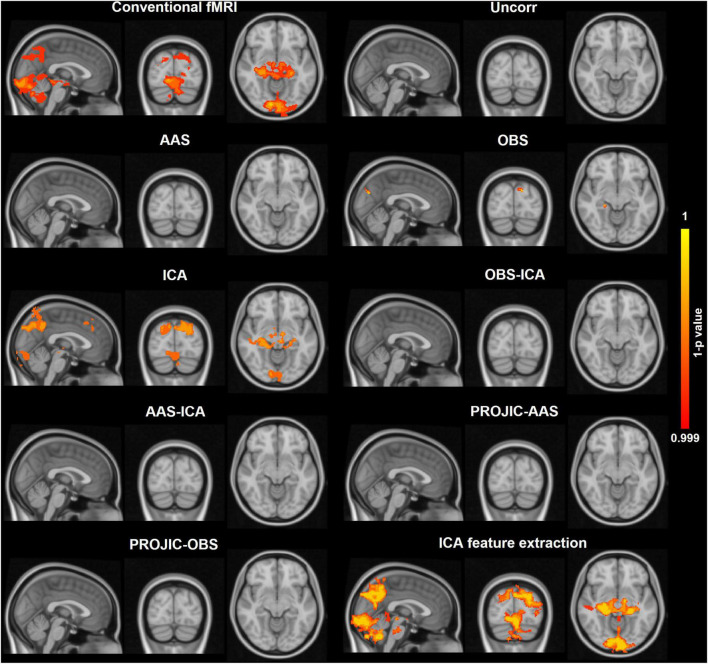
Corrected threshold-free cluster enhancement voxel-wise group-level statistical maps obtained from the EC-EO task fMRI data analysis (*n* = 15) using either the task design or the EEG alpha power fluctuations to generate the blood-oxygen level-dependent (BOLD) signal predictors used in the general linear model. For the conventional fMRI analysis (task design), the map shows the voxels that displayed a positive association with the model (higher BOLD signal in EO vs. EC conditions). For the EEG-informed fMRI analyses, the maps show the voxels where the BOLD signal exhibited a significant negative association with the EEG alpha power derived BOLD signal predictor. A comparison is shown between the maps obtained using the predictors derived from the same EEG signals, corrected using each BCG correction method or IFE. Only IFE preserved the negative relationship between alpha power fluctuations and the BOLD signal, providing very similar maps to those obtained from the conventional fMRI analysis. The color scale shows the 1-p statistical values with a threshold set at *p* < 001.

To evaluate if the BCG artifact biased multimodal data analysis results, we performed EEG-informed fMRI analysis using alpha power fluctuations derived from the EEG-fMRI EC-EO condition to generate the BOLD signal predictors. EEG predictors were obtained from the same EEG-fMRI EC-EO signals corrected with one of the eight approaches (AAS, OBS, ICA, OBS-ICA, AAS-ICA, PROJIC-AAS, PROJIC-ICA, and IFE). The results were compared to those obtained with conventional fMRI analysis, using the task design to build the hemodynamic response model (positive contrast). Figure 7 displays the statistical parametric maps obtained using the task design and each of the EEG-derived predictors. As expected, in the task design predictor maps we observed BOLD signal increases during the EO condition and decreases during the EC condition within occipital and parietal cortices. BOLD signal changes were accurately predicted by EEG signals, as observed in the maps from the EEG-derived predictor generated after using IFE to extract alpha power fluctuations. Importantly, BCG residuals/signal loss that remained after implementing all the tested BCG correction approaches biased the results of the EEG-informed fMRI analysis, obscuring the associations between alpha power and BOLD signal fluctuations.

## 4 Discussion

In this study we aimed to characterize the impact of the BCG artifact on spontaneous EEG spectral power and to compare some of the most popular available BCG correction approaches. Our main focus was to assess the preservation of resting-state EEG spectral properties by statistically comparing the absolute and relative power changes in the EEG data simultaneously acquired with fMRI (corrected with different methods) with respect to the uncorrected data and the data obtained outside of the MR environment. We further investigated whether the functional information from EEG spectral power could be retrieved regardless of the presence of BCG artifact residuals, by evaluating the alpha power reactivity to an EC-EO task. Finally, we wanted to assess how the selection of the BCG artifact correction method influenced the results from EEG-informed fMRI analysis. Although several studies have previously compared different BCG correction methods to assess artifact reduction and signal preservation (Debener et al., 2006; Vanderperren et al., 2010; Marino et al., 2018; Bullock et al., 2021), ours is one of the few studies that: (1) Focus on the preservation of spontaneous brain oscillations rather than ERPs, (2) Characterize the impact of BCG artifact removal using seven state-of-the-art methods by using a specific task paradigm to test the functional reactivity of a particular spontaneous brain rhythm (alpha oscillations), and (3) Provide a direct side-by-side comparison of the impact of using different BCG correction approaches prior to multimodal EEG-informed fMRI analysis.

The uncorrected rs-EEG-fMRI showed a marked increase in absolute power across all frequency bands, more pronounced within the theta and slow beta bands. Relative power was also severely distorted, making uncorrected EEG signals unusable for any analysis purposes. We found that, even though a clear reduction of the artifact was observed on the power spectra of our rs-EEG-fMRI data, none of the BCG artifact removal approaches tested (AAS, OBS, ICA, OBS-ICA, AAS-ICA, PROJIC-AAS, PROJIC-OBS) entirely preserved the spectral profile of EEG signals, due to both artifact residuals and induced EEG signal losses. Overall, in line with previous reports (Srivastava et al., 2005; Debener et al., 2006; Mayeli et al., 2021), we found better results with ICA-based approaches, especially when used after AAS or OBS, as compared to the conventional AAS and OBS and PROJIC approaches. Additionally, large variability in the artifact correction outcomes was observed, with some subjects even showing decreased absolute power compared to their outside rs-EEG, which may be reflecting EEG signal losses after the artifact correction procedure (Ullsperger and Debener, 2010; Marino et al., 2018). To our surprise, the estimation of the individual alpha peak frequency and center of gravity were preserved even in the uncorrected rs-EEG-fMRI data, suggesting that such features can be successfully extracted from EEG data recorded inside the MR environment. We replicated this finding on the Inside rs-EEG data, supporting the robustness of this approach (Klimesch et al., 1990; Corcoran et al., 2018) and suggesting that these features may be extracted from simultaneous EEG-fMRI studies, and could potentially be used as features for integrative analysis.

The severe distortions observed in the absolute and relative spectral power highlight the huge impact BCG artifact residuals have on the resting-state EEG signals and demonstrate that artifact residuals remain after applying all the tested BCG correction methods, impairing the preservation of spontaneous EEG signal properties, as opposed to what is observed in event related potential studies (Debener et al., 2006; Assecondi et al., 2010; Vanderperren et al., 2010). We were also interested in investigating if, despite the generalized distortions of the power spectrum, functional information from task-reactive spontaneous EEG signals could be retrieved. We selected our task considering that alpha power reactivity to eyes closure-opening is one of the most robust phenomena observed in human EEG (Berger, 1929; Adrian and Matthews, 1934; Barry and De Blasio, 2017) and that it is the most commonly used paradigm on simultaneous EEG-fMRI resting-state studies (Goldman et al., 2002; Moosmann et al., 2003; Munck and Maurits, 2006; de Munck et al., 2007). We found that the occipital alpha rhythm reactivity to the EC-EO task was retrieved when using IFE and, to a lesser extent, with the OBS-ICA, and AAS-ICA–based corrections. However, neither the AAS, OBS, ICA or the PROJIC-AAS and PROJIC-OBS approaches preserved a clear distinction between EC and EO states.

Considering the potential implications of our findings, we then evaluated how the choice of the BCG correction method impacted the generation of EEG alpha power derived BOLD signal predictors used for EEG-informed fMRI analysis. Only the data processed using IFE of the alpha rhythm showed a clear significant inverse relation between alpha power and the BOLD signal from the occipital and parietal cortices, yielding similar statistical parametric maps to those obtained with conventional fMRI analysis (Figure 7), and those reported in previous alpha power EEG-informed fMRI studies (Goldman et al., 2002; Laufs et al., 2003; Moosmann et al., 2003; de Munck et al., 2007). None of the seven BCG removal methods tested here allowed to preserve the EEG alpha fluctuations to the same extent, and no statistical associations with the BOLD signal were observed in the EEG-informed fMRI analysis. These results provide compelling evidence that BCG artifact residuals and/or EEG signal losses related to the artifact removal procedure severely impair data quality and mask the functional association between EEG alpha power and occipito-parietal BOLD signal, hampering our interpretations from multimodal EEG-fMRI integrative analyses (Goldman et al., 2002; Scrivener, 2021; Warbrick, 2022).

Overall, our results demonstrate that state-of-the-art BCG artifact correction approaches still have important limitations. Our work highlights the need for refining and standardizing existing methods, and to develop novel approaches to deal with the BCG artifact to fully benefit from the advantages provided by simultaneous EEG-fMRI. We also highlight the need to validate current and novel approaches by evaluating the preservation of spontaneous EEG brain rhythms and their impact on multimodal integrative analyses. We demonstrated that IFE was effective to rescue the alpha rhythm reactivity to the eyes closure-opening task, though future studies should design specific paradigms to test the reactivity of other brain rhythms.

Regarding new software implementations, interesting proposals have arisen among the EEG-fMRI community. Given that the delay between the ECG and the BCG peak may vary over time (Oh et al., 2014), the adaptative OBS method was proposed to improve the results obtained with conventional OBS, by adjusting the variable delay between the QRS peak and the main BCG artifact peak (Marino et al., 2018). Another set of promising alternatives are the machine learning-based approaches that employ different data learning algorithms to better identify and classify the BCG artifact (Abolghasemi and Ferdowsi, 2015; McIntosh et al., 2021; Ebrahimzadeh et al., 2022; Lin et al., 2022). Even with the development of new signal processing tools that allow to better characterize and correct the BCG artifact, it has become evident that the solution for the BCG artifact problem must come not from software but most likely from hardware-based approaches, that incorporate additional elements or change the configuration of the EEG-fMRI setup to measure and/or reduce the artifact during data acquisition (Ullsperger and Debener, 2010; Jorge et al., 2014; Ebrahimzadeh et al., 2022). Promising examples include modified EEG caps containing a reference adapting layer (Xia et al., 2014) or carbon-based wire loops (van der Meer et al., 2016) that record electrode motion and use this information to better model and subtract the BCG artifact from the data, and also modifications in the materials for electrodes and leads as well are their geometrical configuration (Chowdhury et al., 2015; Assecondi et al., 2016).

Our study also contributes to the field by providing a simple, easy-to-implement workflow to characterize the impact of the BCG artifact and assess the efficiency of BCG artifact removal methods to reduce the artifact and preserve EEG spectral properties, which may be useful when attempting to validate novel BCG artifact correction approaches in resting-state EEG data or implementing an EEG-fMRI protocol in a new facility. Also, by making our dataset available to the scientific community we hope to incentivize other groups to participate in EEG-fMRI research and take advantage of these data to explore and validate novel BCG removal approaches, aiming to increase the collective effort to solve this 30-year-old problem in the field of simultaneous EEG-fMRI.

### 4.1 Study limitations

The present study has many strengths as it is one of the few works characterizing the preservation of spectral properties of resting-state EEG and EEG reactivity to a task after BCG artifact correction, and its impact on multimodal EEG-informed fMRI analysis. We carefully selected a sample of young healthy adults to assess EEG data quality. Although the number of subjects was relatively small (*N* = 20), it is much higher than many previous studies assessing EEG data quality during simultaneous EEG-fMRI experiments. Additionally, we validated and replicated our main findings in the rs-EEG-fMRI data by also analyzing the data recorded inside the scanner without fMRI acquisition. Overall, we found very similar results, supporting the idea that GA residuals do not influence our results from the EEG-fMRI data and showing that there was a consistent pattern between the results obtained from two independent sets of data from the same subjects, further supporting our conclusions.

Several limitations should also be considered. For practical reasons, the outside-EEG was always recorded before the inside-EEG and simultaneous EEG-fMRI for all subjects. Not counterbalancing the conditions may bias EEG quantitative measures if the subject’s mental state has changed due to vigilance fluctuations or fatigue. However, given that in this study the time between conditions was relatively short (around 15 min between outside and inside scanner EEG recordings) we do not expect this to significantly affect our findings. Additionally, only male subjects were included in this study, which impacts the generalizability of our results and poses the need of replicating these findings in a cohort of female participants. Although we put great effort into matching the experimental conditions across subjects, we also acknowledge that our results may be influenced by the subject’s head position relative to the B0 magnetic field and the amount of movement during the recordings (Debener et al., 2008; Yan et al., 2010; Mullinger et al., 2013b), both of which increase the within and between-subject variability in the BCG artifact spectral profile.

Inconsistent results as compared to other studies may be attributed to differences in methodologies, such as the use of low-impedance, conductive paste EEG caps in other studies (Debener et al., 2008; Vanderperren et al., 2010; Mullinger et al., 2013b; Arrubla et al., 2014), which may have some advantages over high-impedance caps as the one used here, but also differ in terms of the length and geometrical arrangement of the EEG wires and placement of the EEG amplifier relative to the B0 magnetic field (Chowdhury et al., 2015; Assecondi et al., 2016). Our data also suggest that cardiac signal recording using conventional ECG montages is not ideal for EEG-fMRI studies, and therefore other measurements of cardiac activity (or ideally scalp measurements of the BCG artifact itself) should be used, given that low quality ECG data may result in a poor estimation of the QRS-peak, which impacts the efficiency of the BCG artifact correction process (Iannotti et al., 2014). Another aspect to consider is that the parameter tunning for each BCG correction approach may dramatically influence the results. Both AAS and OBS were implemented using a fixed delay between the QRS events and the BCG amplitude peak (210 ms) which actually has been proved to be very variable within and between individuals (Yan et al., 2010; Marino et al., 2018). The number of principal components used to implement the OBS-based correction approach was kept constant for all subjects, while some studies have shown that optimizing parameters for each subject improves the results from the artifact correction process (Abreu et al., 2016; Marino et al., 2018). The parameters used here were selected to match the default options of the fMRIB toolbox, which are also the parameters typically used in many EEG-fMRI studies. We should therefore keep in mind that there may be room for improving the artifact correction procedure by fine-tuning these parameters (Marino et al., 2018). For ICA–based corrections, the ICs corresponding to the BCG artifact were manually selected. Although we used standard criteria to select the artifact-related components this generates a potential bias, and future studies should try using automatic or semi-automatic independent component selection algorithms. It is also very plausible that having a higher number of electrodes would improve the spatial characterization of the artifact, facilitating the selection of components and improving signal preservation. The most recent PROJIC approaches (Abreu et al., 2016) might significantly improve by adjusting different parameters on an individual subject basis. Here we used the recommended parameters across all individuals, and therefore this question should be addressed in future studies.

Finally, we evaluated the preservation of EEG functional properties only by focusing on posterior alpha power reactivity to the EC-EO task. Although we demonstrated that ICA feature extraction allowed to retrieve the associations between alpha power and hemodynamic signals, future studies are needed to evaluate if other spontaneous brain rhythms can be preserved, especially considering that lower frequencies are even more affected by the BCG artifact harmonic frequencies, and that higher frequencies have a much lower amplitude compared to the artifact waveforms. To tackle this question, other study designs need to be implemented to evaluate the reactivity of these particular rhythms (i.e., cognitive tasks, spontaneous activity recording in other natural physiological states such as sleep).

## 5 Conclusion

Overall, our study provides strong evidence that the most commonly used BCG correction methods have important limitations and were not able to entirely preserve the spectral power features of resting-state eyes-closed EEG activity (excepting for the individual alpha peak frequency and center of gravity), nor the functional reactivity of EEG signals to a simple EC-EO task, using this particular EEG-fMRI setup. Importantly, the EEG signal distortions compromised the results from integrative multimodal data analysis, evidencing the imposed difficulty of reliably studying the relationship between spontaneous electrophysiological activity and hemodynamic brain responses without optimal EEG data quality. ICA feature extraction allowed to preserve EEG oscillations related to the EC-EO task and to obtain reliable predictors for EEG-informed fMRI analysis. Future studies assessing novel or adapted hardware and software strategies to deal with the BCG artifact are needed and should be validated by assessing the preservation of EEG signal properties as the main concern.

## Data availability statement

The datasets presented in this study can be found in online repositories. The names of the repository/repositories and accession number(s) can be found below: https://data.mendeley.com/datasets/crhybxpdy6.

## Ethics statement

The studies involving human participants were reviewed and approved by the Bioethics Committee of the Instituto de Neurobiología, Universidad Nacional Autónoma de México, Campus Juriquilla. The patients/participants provided their written informed consent to participate in this study. Written informed consent was obtained from the individual(s) for the publication of any potentially identifiable images or data included in this article.

## Author contributions

JG-R: conceptualization, methodology, software, validation, formal analysis, investigation, resources, data curation, writing – original draft, editing and review, visualization, and project administration. MC-C: conceptualization, methodology, validation, formal analysis, writing – review and editing, and supervision. LC: conceptualization, methodology, validation, investigation, resources, writing–review and editing, and funding acquisition. JR-G: conceptualization, methodology, and validation. EP-A: conceptualization, methodology, validation, investigation, resources, data curation, supervision, project administration, and funding acquisition. All authors contributed to the article and approved the submitted version.
