# Supplementary material for: Preservation of EEG spectral power features during simultaneous EEG-fMRI
Source: Front Neurosci. 2022 Dec 23;16:951321. doi: 10.3389/fnins.2022.951321 (PMC9816433; doi:10.3389/fnins.2022.951321)
Supplement: Supplementary file 1 [file Data_Sheet_1.docx]

Supplementary Material

# Supplementary Figures and captions

**Supplementary Figure 1.**


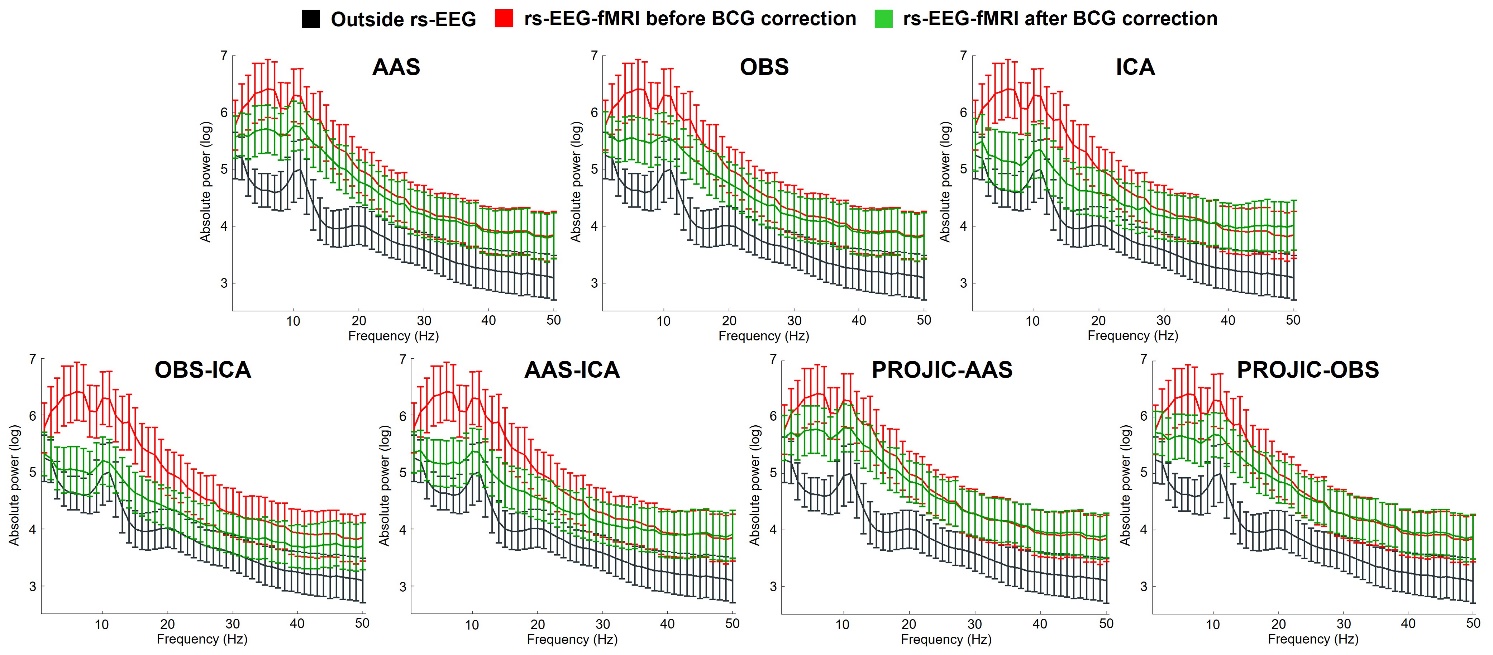


**Supplementary Figure 1.** Average power spectrum (and standard deviation) computed from the resting-state eyes-closed EEG signal of all electrodes from all subjects. Each panel shows a comparison between the Outside rs-EEG (black, repeated in all panels) and the Inside rs-EEG before (red, repeated in all panels) and after (green) correcting the BCG artifact with each of the seven methods: AAS, OBS, ICA, OBS-ICA, AAS-ICA, PROJIC-AAS and PROJIC-OBS. Consistent with the results from the rs-EEG-fMRI data, ICA-based corrections performed better in reducing the BCG artifact contribution and preserving the Inside rs-EEG spectral profile, though power remained higher than the outside rs-EEG. The number of components removed per each method (mean; sd; range) was 8.8; 1.5; 7-12 for ICA, 5.5; 1.4; 3-9 for OBS-ICA and 4.5; 1.1; 3-6 for AAS-ICA.

**Supplementary Figure 2.**


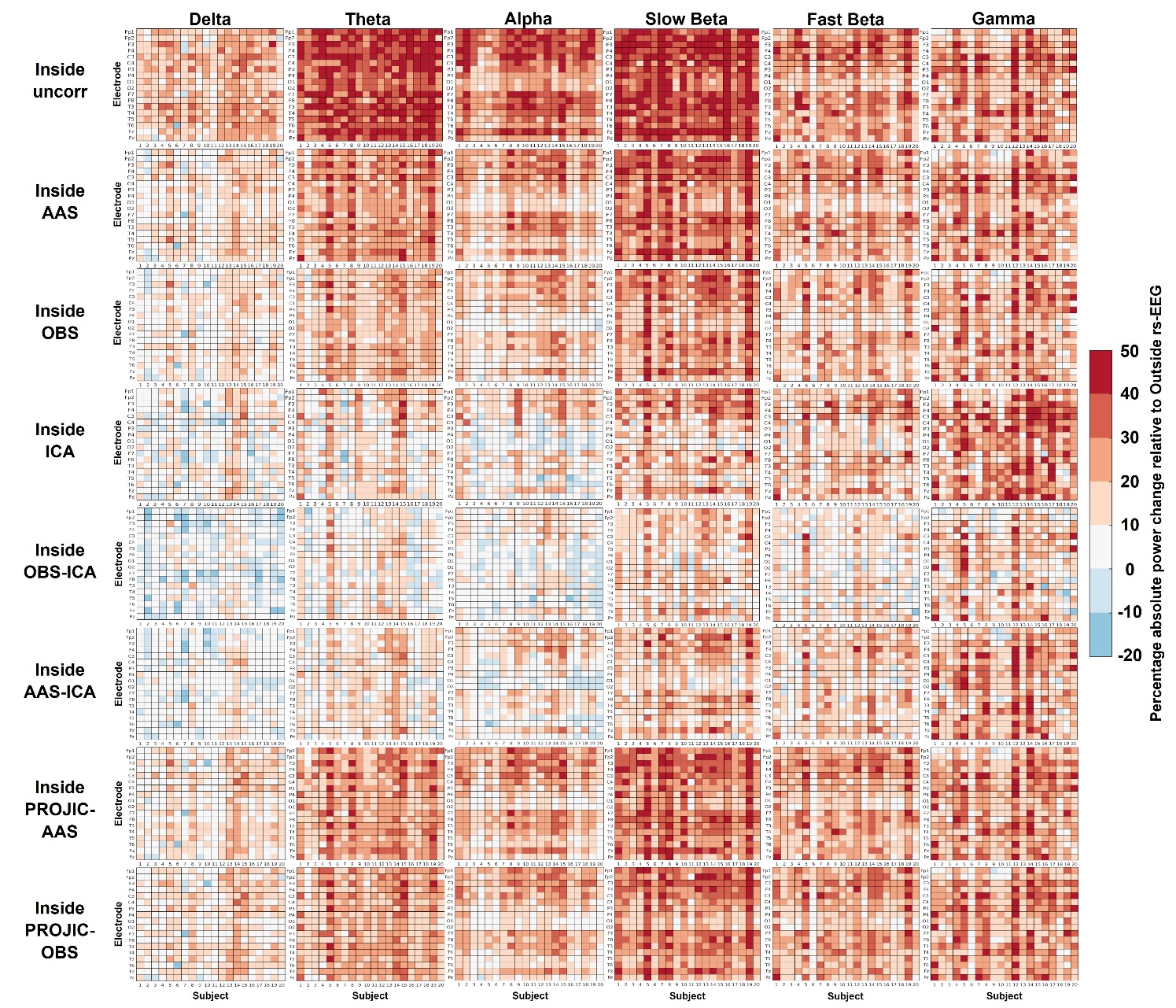


**Supplementary Figure 2.** Percentage change (colorbar) in the absolute power of each frequency band of the Inside rs-EEG data before and after BCG artifact removal, relative to the Outside rs-EEG. Each matrix shows all electrodes (rows) for each subject (columns). A negative percentage indicates lower absolute power in the Inside rs-EEG compared to the Outside rs-EEG. Consistent with the results from the rs-EEG-fMRI data, ICA-based corrections performed better in reducing the BCG artifact contribution and preserving the Inside rs-EEG spectral profile (especially for delta, theta, and alpha bands), though artifact residuals and/or absolute power decreases were evident for most subjects, across all frequency bands.

**Supplementary Figure 3.**


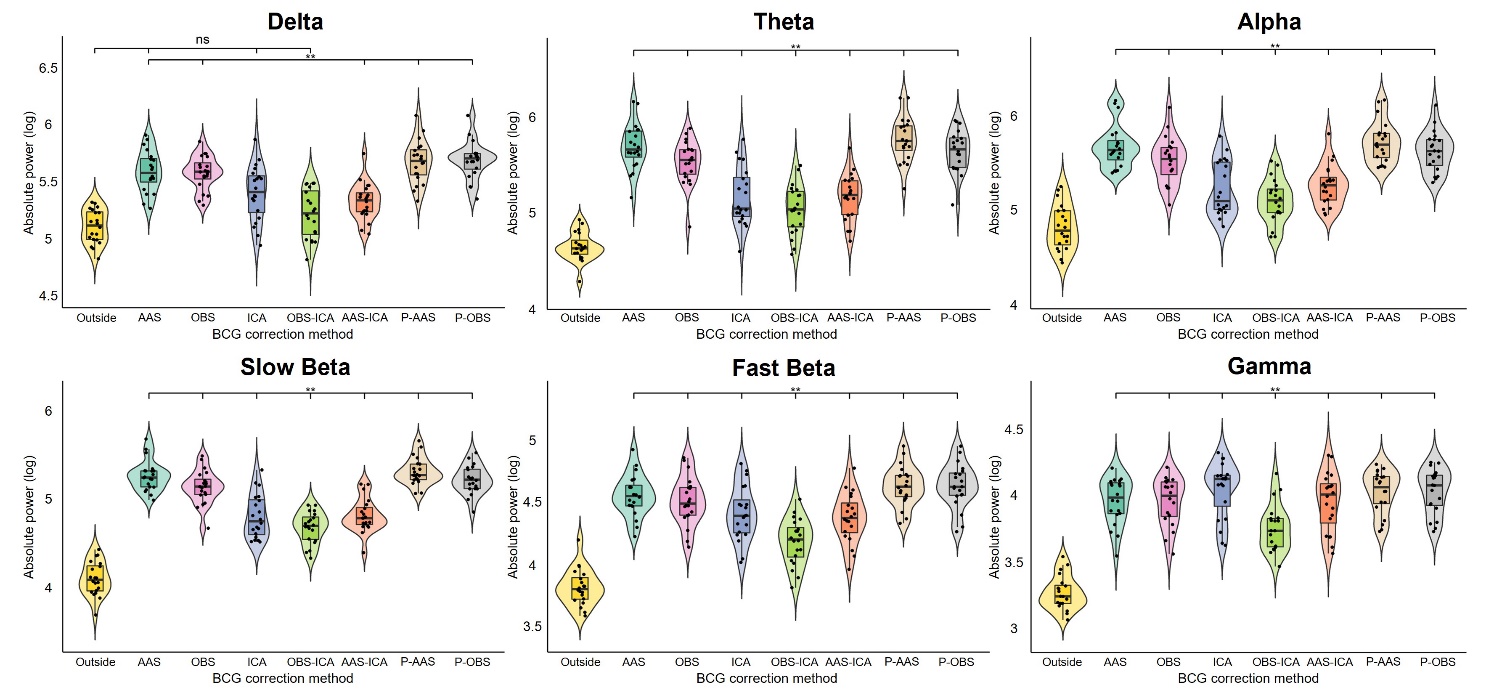


**Supplementary Figure 3.** Results of the repeated measures ANOVAs comparing the average absolute power of all electrodes from all subjects between the Outside rs-EEG and the Inside rs-EEG data corrected using each of the seven BCG removal methods. Each frequency band was analyzed separately. The asterisks indicate significant statistical differences (*p*adj < 0.05) between the corrected Inside rs-EEG and the Outside rs-EEG data. Consistent with the results from the rs-EEG-fMRI data, a generalized increase in absolute power across all frequency bands was observed for the data recorded inside the scanner, which remained significant after applying all BCG correction methods. Note that PROJIC-AAS and PROJIC-OBS were abbreviated as P-AAS and P-OBS, respectively.

**Supplementary Figure 4.**


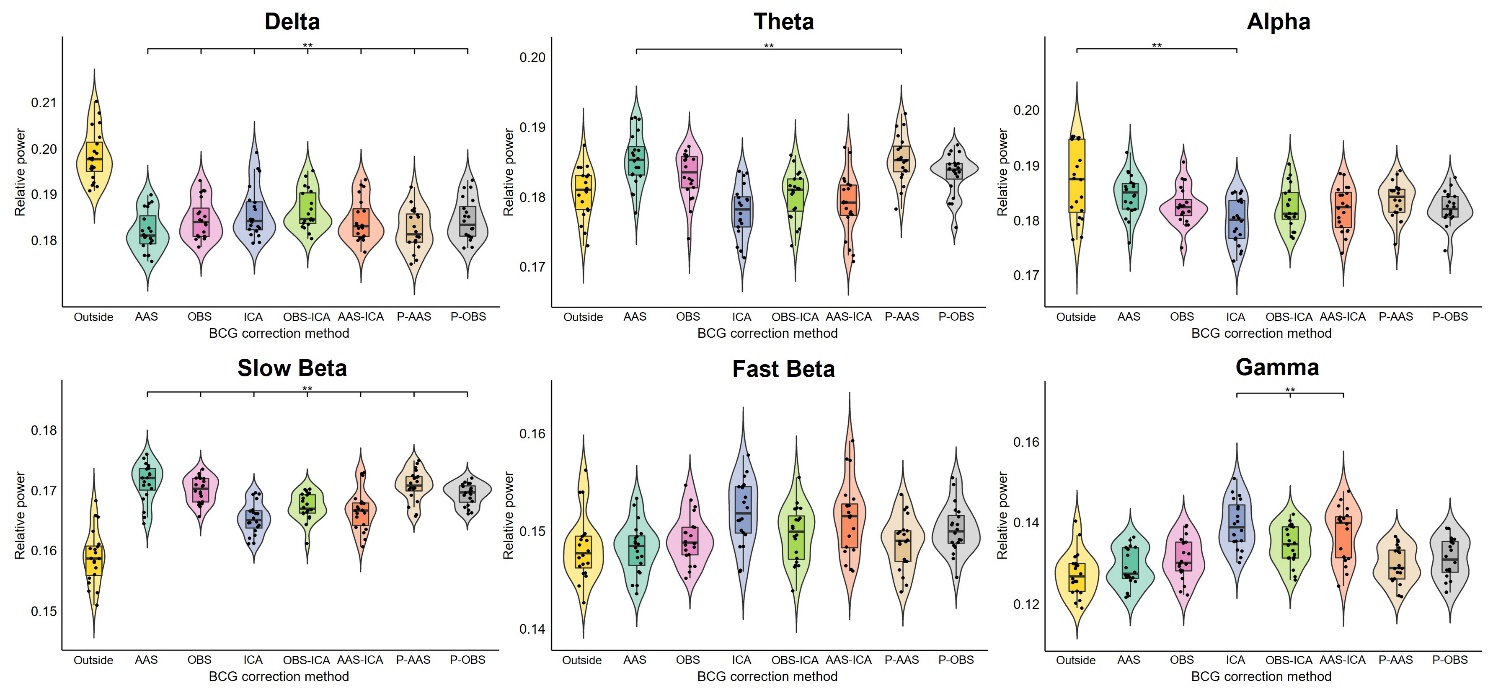


**Supplementary Figure 4.** Results of the repeated measures ANOVAs comparing the average relative power of all electrodes from all subjects between the Outside rs-EEG and the Inside rs-EEG data corrected using each of the seven BCG removal methods. Each frequency band was analyzed separately. The asterisks indicate significant statistical differences (*p*adj < 0.05) between the corrected Inside rs-EEG and the Outside rs-EEG data. Consistent with the results from the rs-EEG-fMRI data, relative power was altered across all frequency bands for the data recorded inside the scanner. Some correction approaches rescued relative power for some frequency bands, but the overall spectral power profile remained altered across all BCG correction methods. Note that PROJIC-AAS and PROJIC-OBS were abbreviated as P-AAS and P-OBS, respectively.

**Supplementary Figure 5.**


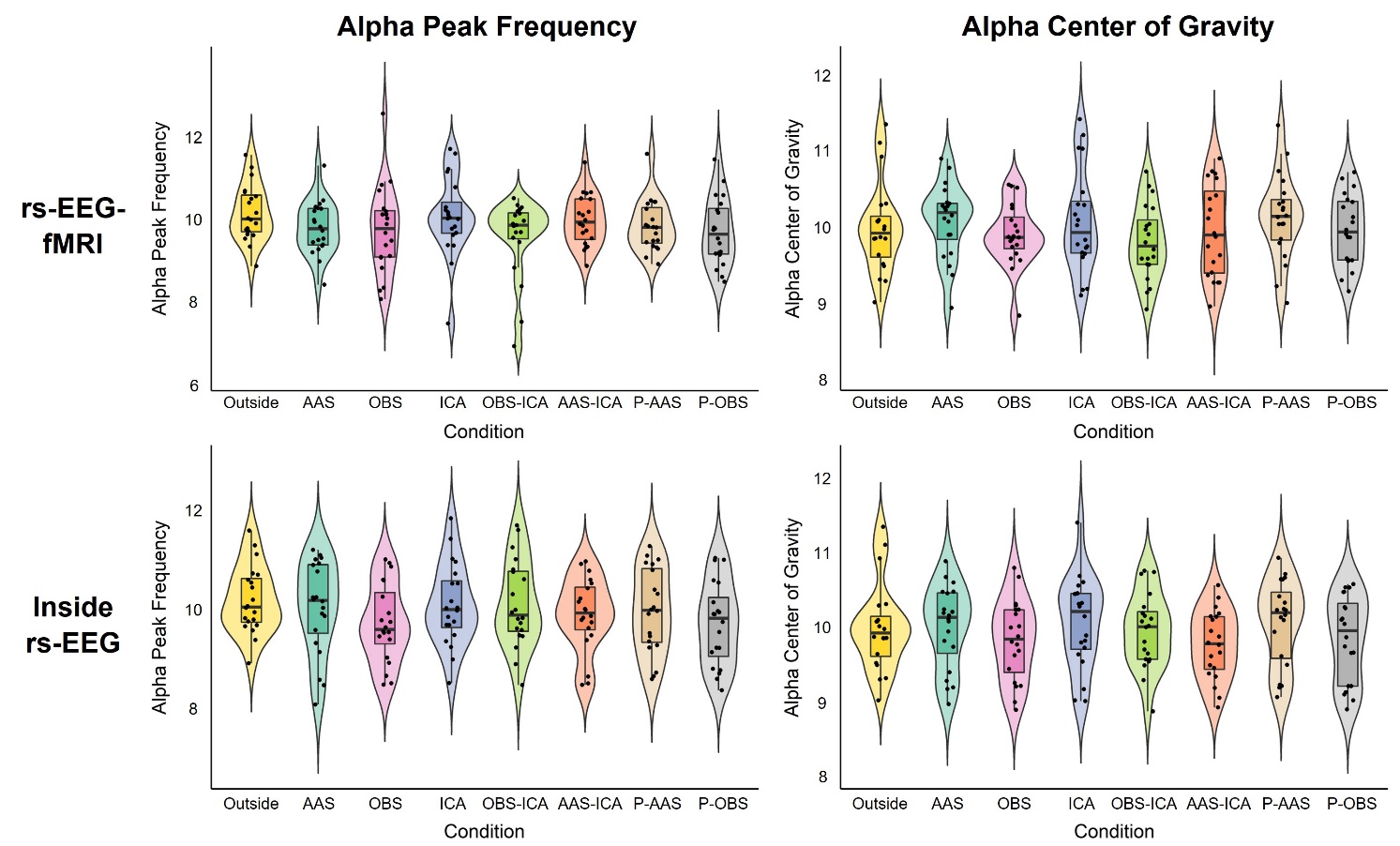


**Supplementary Figure 5.** Results of the repeated measures ANOVAs comparing the individual peak alpha frequency and alpha center or gravity between the Outside rs-EEG and the rs-EEG-fMRI/Inside rs-EEG data corrected using each of the seven BCG removal approaches. No significant statistical differences (p_adj_ < 0.05) were found between the estimations of the individual peak alpha frequency and the alpha center of gravity of the corrected rs-EEG-fMRI/Inside rs-EEG signals and the Outside rs-EEG data. Note that PROJIC-AAS and PROJIC-OBS were abbreviated as P-AAS and P-OBS, respectively.

**Supplementary Table 1.**

|  |  | **Out-side** | **Un-corr** | **AAS** | **OBS** | **ICA** | **OBS-ICA** | **AAS-ICA** | **P-AAS** | **P-OBS** |
| --- | --- | --- | --- | --- | --- | --- | --- | --- | --- | --- |
| **rs-EEG-fMRI** | **Mean nchan (PAF)** | **14.7** | **13.45** | **11.4** | **8.9** | **12.2** | **11.4** | **12.7** | **11.6** | **11.3** |
|  | **SD nchan (PAF)** | **2.1** | **2.7** | **2.7** | **3.2** | **3.5** | **4** | **3.2** | **3.1** | **3.5** |
|  | **Mean nchan (CoG)** | **17.3** | **16.9** | **17** | **16.5** | **17.4** | **17.3** | **17.3** | **16.8** | **16.9** |
|  | **SD nchan (CoG)** | **1.4** | **1.6** | **1.5** | **1.8** | **1** | **1.1** | **1.1** | **1.4** | **1.7** |
| **Inside rs-EEG** | **Mean nchan (PAF)** | **14.7** | **13.1** | **12.5** | **8.1** | **12.6** | **10.2** | **11.7** | **12.8** | **12.6** |
|  | **SD nchan (PAF)** | **2.1** | **2.5** | **3.5** | **3.5** | **4.1** | **3.7** | **2.5** | **3.8** | **2.9** |
|  | **Mean nchan (CoG)** | **17.3** | **16.7** | **16.8** | **16.8** | **17.2** | **16.9** | **16.8** | **17** | **16.8** |
|  | **SD nchan (CoG)** | **1.4** | **1.5** | **1.8** | **2** | **1.5** | **1.8** | **2** | **1.7** | **1.6** |

**Supplementary table 1.** Mean and standard deviation of the number of electrodes used for computing the individual peak alpha frequency and alpha center of gravity from the Outside rs-EEG and the rs-EEG-fMRI/Inside rs-EEG data. Note that PROJIC-AAS and PROJIC-OBS were abbreviated as P-AAS and P-OBS, respectively.
